# Supplementary material for: Highly robust and soft biohybrid mechanoluminescence for optical signaling and illumination
Source: Nat Commun. 2022 Jul 7;13:3914. doi: 10.1038/s41467-022-31705-6 (PMC9263131; doi:10.1038/s41467-022-31705-6)
Supplement: Supplementary file 1 — Supplementary Information [file 41467_2022_31705_MOESM1_ESM.pdf]

Supplementary Information for

Highly robust and soft biohybrid mechanoluminescence for optical signaling and illumination

Chenghai Li[1], Qiguang He[1], Yang Wang[2], Zhijian Wang[1], Zijun Wang[2], Raja Annapooranan[2], Michael I. Latz[3], Shengqiang Cai[1, 2, *]

[1] Department of Mechanical and Aerospace Engineering, University of California, San Diego, La Jolla, CA 92093, USA.
[2] Materials Science and Engineering Program, University of California, San Diego, La Jolla, CA 92093, USA.
[3] Scripps Institution of Oceanography, University of California, San Diego, La Jolla, CA 92093, USA.
* Correspondence and requests for materials should be addressed to S.C. (Email: shqcai@ucsd.edu).

This PDF file includes:

Supplementary Figures 1 to S21
Supplementary Notes 1 to 4
Legends for Supplementary Movies 1 to 9
Supplementary References

Other supplementary materials for this manuscript include the following:

Supplementary Movies 1 to 9

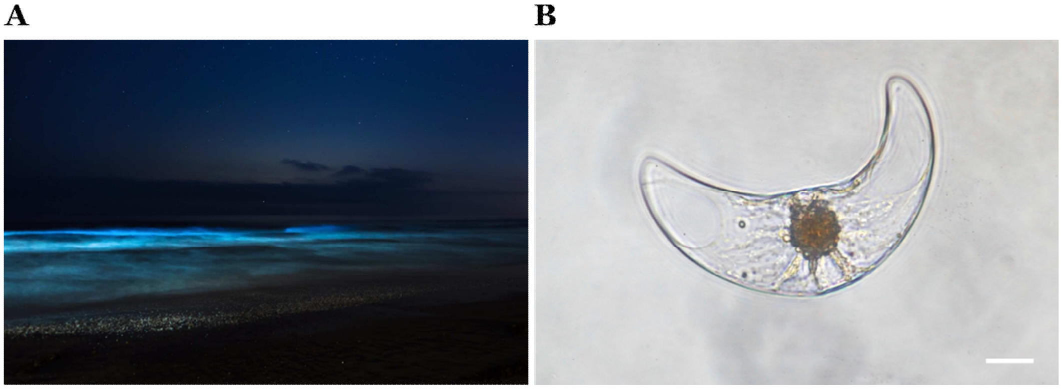

Supplementary Figure 1. Ocean bioluminescence and microscope image of the unicellular marine dinoflagellate *P. lunula*. (**A**) Bioluminescent ocean waves along the beach of La Jolla, San Diego, California, caused by bioluminescent dinoflagellates responding to fluid shear stress. Photo credit to Dr. Min Tang; used with permission. (**B**) *P. lunula* has a typical size of ∼40 μm in width and ∼130 μm in length. The cell is mostly transparent, except for the prominent nucleus. Bioluminescence is emitted from vesicles in the vicinity of the nucleus. Scale bar, 20 μm.

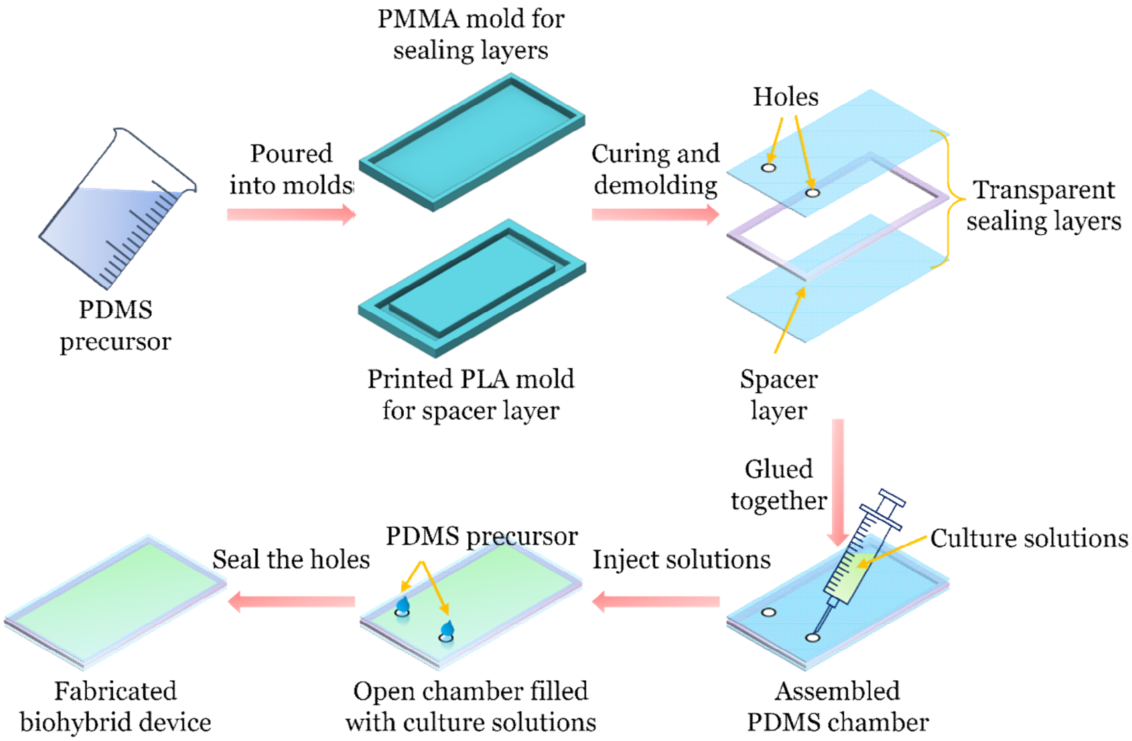

Supplementary Figure 2. Fabrication process of the biohybrid devices. The PDMS precursor was mixed, degassed, and then poured into a 3D printed PLA mold and a laser cut PMMA mold for making the spacer layer and sealing layers, respectively. After curing, we punched two holes on one sealing layer to allow the injection of dinoflagellate culture solution. Then, the spacer layer and two sealing layers were glued together with the PDMS precursor to form a PDMS chamber. The dinoflagellate culture solution was firstly transferred into a plastic syringe and then injected into the PDMS chamber through the holes. Bubbles were eliminated by repeating this injection process until most bubbles escaped from the elastomer chamber. Finally, the holes were sealed by the PDMS precursor. Fabrication of biohybrid devices with various geometries followed these steps but using different molds. Note that the culture medium was not refreshed in the current study once the biohybrid device was fabricated to form a closed system.

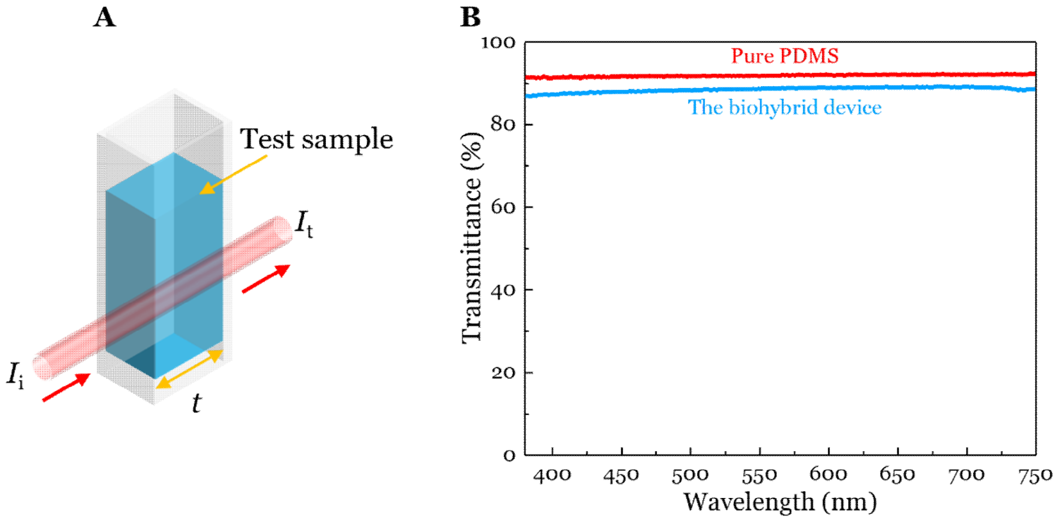

Supplementary Figure 3. Light transmittance of pure PDMS and the biohybrid device. (**A**) Schematic for the measurement of transmittance, defined as I_t/I_i, where I_t is the intensity of the transmitted light, and I_i is the intensity of the incident light. For pure PDMS, the thickness t was 1 mm. For the biohybrid device, the thickness of each layer (two sealing layers and one spacer layer) was 1 mm. Thus, the total thickness t of the biohybrid device filled with the culture solution was 3 mm. (**B**) Spectral dependence of transmittance for pure PDMS and the biohybrid device. Both pure PDMS and the biohybrid device exhibited high transmittance in the visible light range.

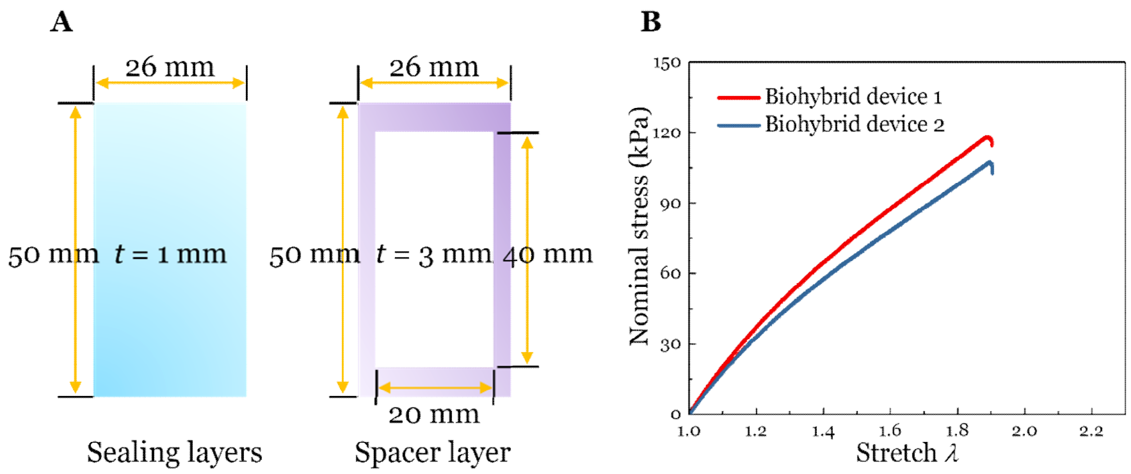

Supplementary Figure 4. Description of the biohybrid device for uniaxial tension tests. (**A**) Schematic showing dimensions of the sealing and spacer layers. (**B**) Nominal stress-stretch curves for the biohybrid devices.

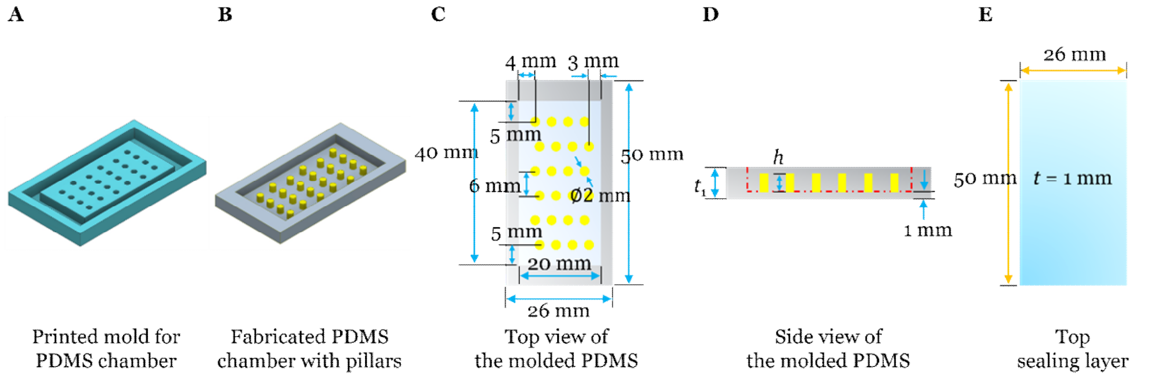

Supplementary Figure 5. Description of the biohybrid device with cylindrical pillars. (**A**) Schematic of the mold for fabricating the PDMS chamber with pillars. (**B**) Schematic of the molded PDMS chamber with pillars. (**C**) The top view and dimensions of the molded PDMS chamber in (B). (**D**) Side view and dimensions of the molded PDMS chamber in (B). The thickness of the molded structure and the height of pillars are t_1 and h, respectively, which are tuned to ensure the total volume of internal chamber is the same as the design without pillars. When h equaled 1.5 mm, 2 mm, and 2.5 mm, the corresponding t_1 was 4.15 mm, 4.2 mm and 4.24 mm, respectively. (**E**) Dimensions of the top sealing layer.

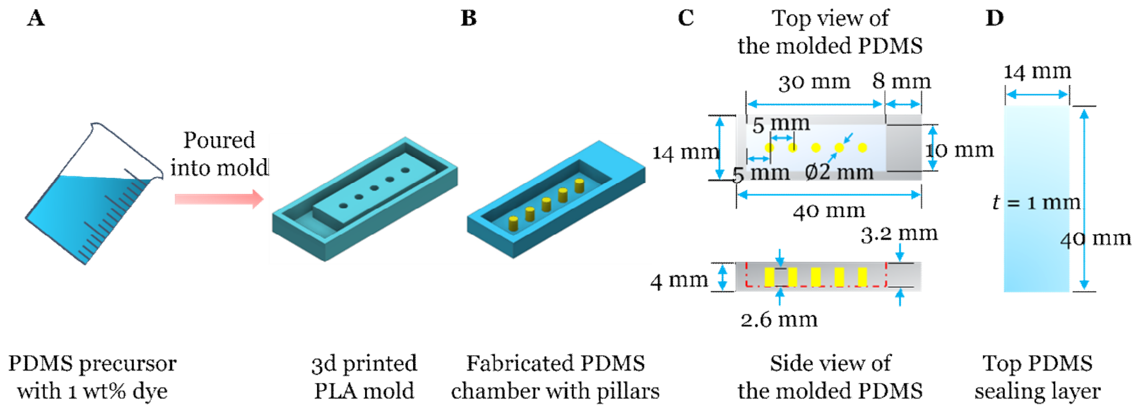

Supplementary Figure 6. Description of the biohybrid device for extension of the color range. (**A**) PDMS precursor with 1% weight:weight colored dye was mixed, degassed and then poured into a 3D printed PLA mold with cylindrical pillars. (**B**) Schematic of the molded PDMS chamber with pillars. (**C**) Top view, side view, and dimensions of the molded PDMS chamber in (B). (**D**) Dimensions of the top sealing layer.

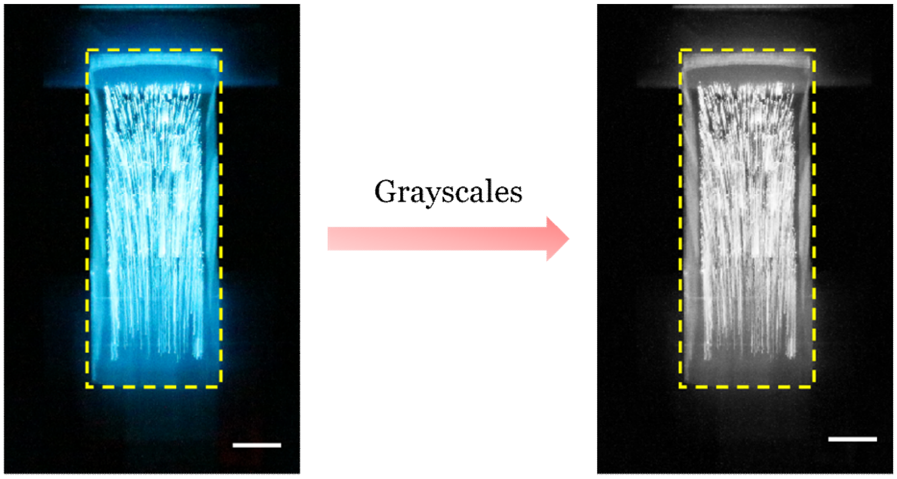

$$Light\ intensity = \frac{\Sigma\, Grayscales\ of\ the\ selected\ area}{Number\ of\ pixels\ of\ the\ selected\ area}$$

Supplementary Figure 7. Definition of light intensity based on grayscale. The light intensity in grayscale units was calculated based on image analysis using MATLAB. The color image was first converted to grayscale format, and then an area outlining the boundary of the sample was selected. Light intensity was defined as the summation of the grayscale values divided by the number of pixels within the selected area.

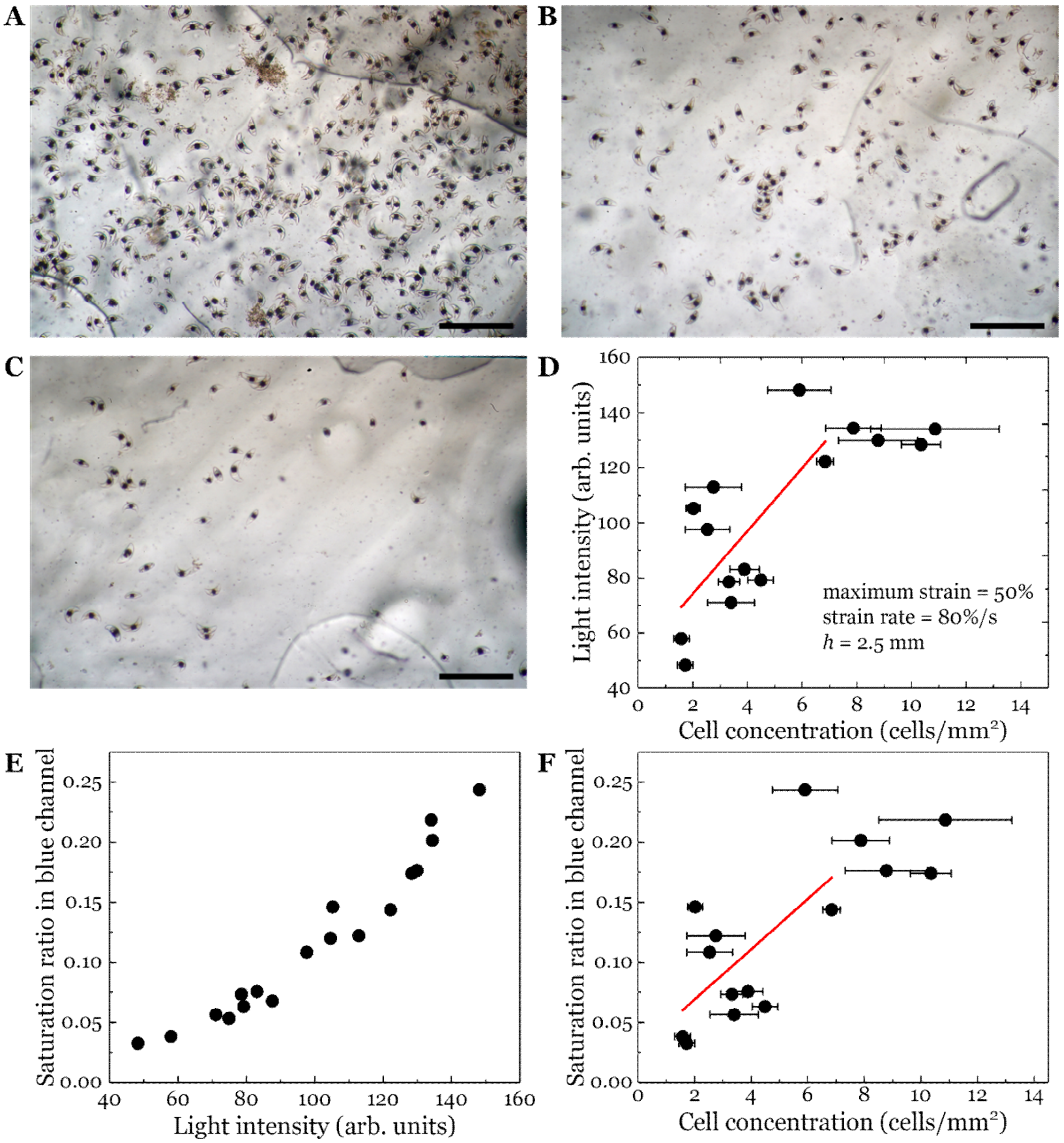

Supplementary Figure 8. Effect of cell concentration on light intensity and saturation ratio in the blue channel of the RGB image. It's noted that chambers with h = 2.5 mm pillars were used. Microscope images of the devices containing *P. lunula* at different concentrations: (**A**) 13.5 cells mm^{-2}, (**B**) 4.8 cells mm^{-2}, and (**C**) 1.6 cells mm^{-2}. Scale bars are 1 mm. (**D**) Light intensity increased with cell concentration up to 6.8 cells mm^{-2} (R^2 = 0.43) and then reached a plateau. (**E**) The saturation ratio in the blue channel of the RGB image increased with light intensity based on grayscale analysis. (**F**) The saturation ratio in the blue channel of the RGB image increased with cell concentration up to 6.8 cells mm^{-2} (R^2 = 0.33) and then reached a plateau. Values in (**D**) and (**F**) represent averages with standard deviations for N = 3 replicates.

Supplementary Note 1

Correlation Between the Concentration of Dinoflagellate Cells and Measured Light Intensity

We first examined the correlation between the concentration of dinoflagellate cells and measured light intensity from the images of bioluminescence (Supplementary Fig. 8). Elastomeric chambers with $h = 2.5$ mm pillars were infused with various concentrations of dinoflagellate cells and then tested under the same loading (Supplementary Fig. 8A-C). A concern with quantifying light emission from color images is the problem of pixel saturation for bright images. We determined that light emission exhibited a plateau for cell concentrations > 6.8 cells mm^{-2} (Supplementary Fig. 8D), due to pixel saturation in the blue channel (Supplementary Fig. 8E, F). Recognizing that pixel saturation in the blue channel is a limitation of quantifying color images, the values presented for light emission in this study are approximations; it is recommended that future studies quantifying light emission use high pixel resolution cameras or sensitive light detectors such as photomultipliers to avoid the pixel saturation issue.

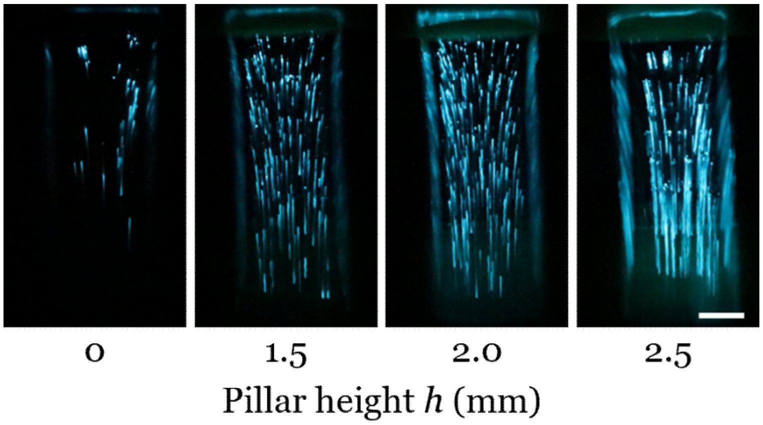

Supplementary Figure 9. Effect of pillar height *h* on bioluminescence induced by stretching. Bioluminescence increased with increasing pillar height *h*, due to enhanced fluid disturbance by taller pillars. A pillar height *h* of 0 mm refers to the chamber without internal pillars. Scale bar, 1cm.

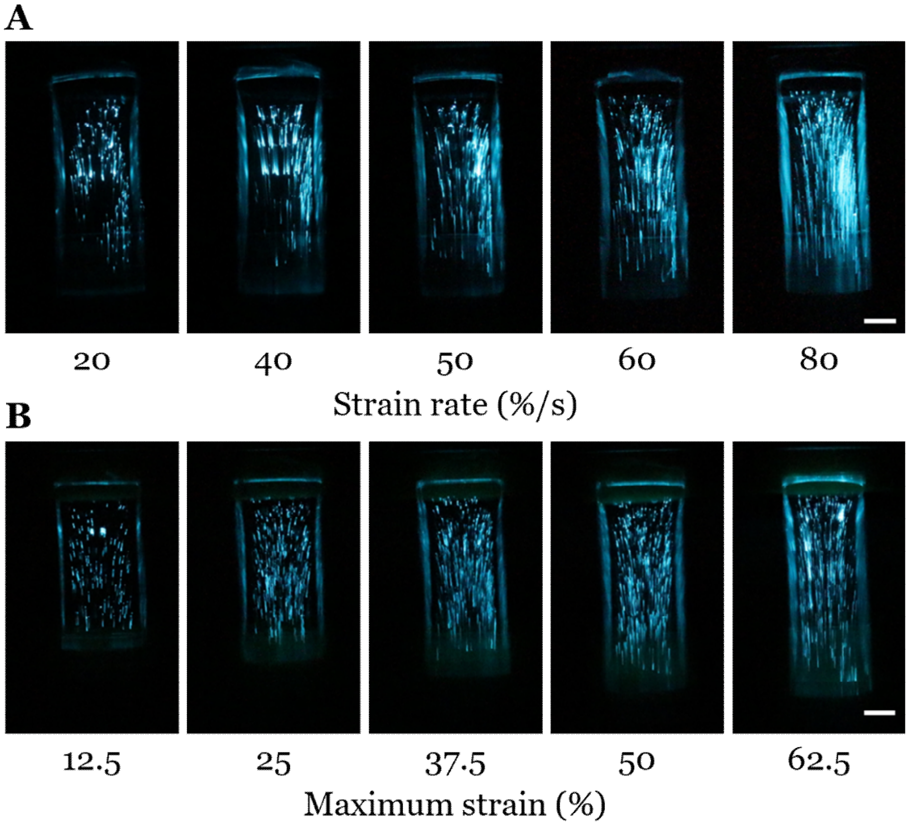

Supplementary Figure 10. Effect of strain rate and maximum strain on bioluminescence for the chamber with *h* = 2.5 mm pillars. (**A**) Light intensity increased with strain rate when the maximum strain was fixed as 50%. (**B**) Light intensity increased with the maximum strain when the strain rate was fixed as 50% s^{-1}. Scale bars, 1 cm.

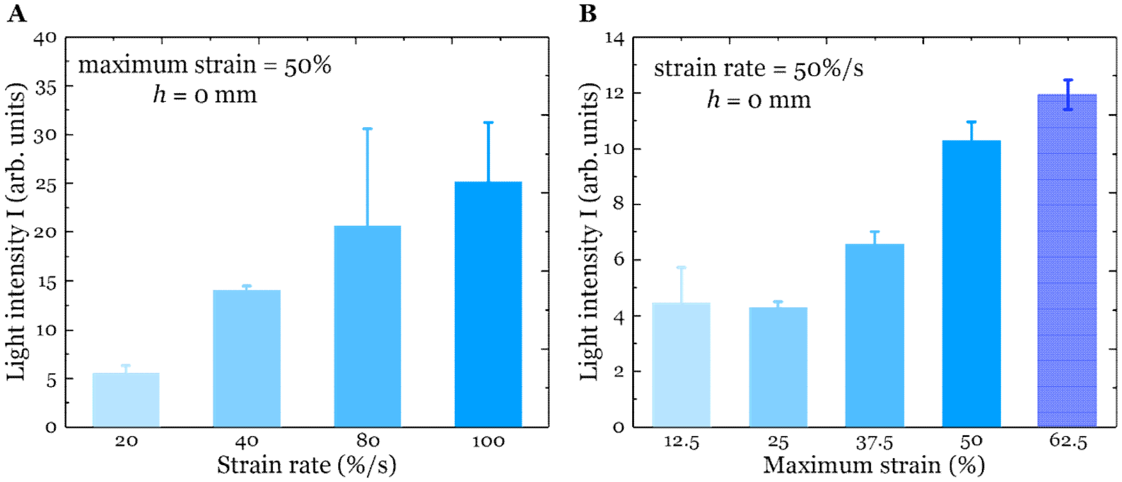

Supplementary Figure 11. Effect of strain rate and maximum strain on the light intensity for the chamber without pillars. (**A**) Light intensity increased with strain rate when the maximum strain was fixed as 50%. (**B**) Light intensity increased with the maximum strain when the strain rate was fixed as 50% s^{-1}. Values in (**A**) and (**B**) represent averages with standard deviations for $N = 3$ replicates.

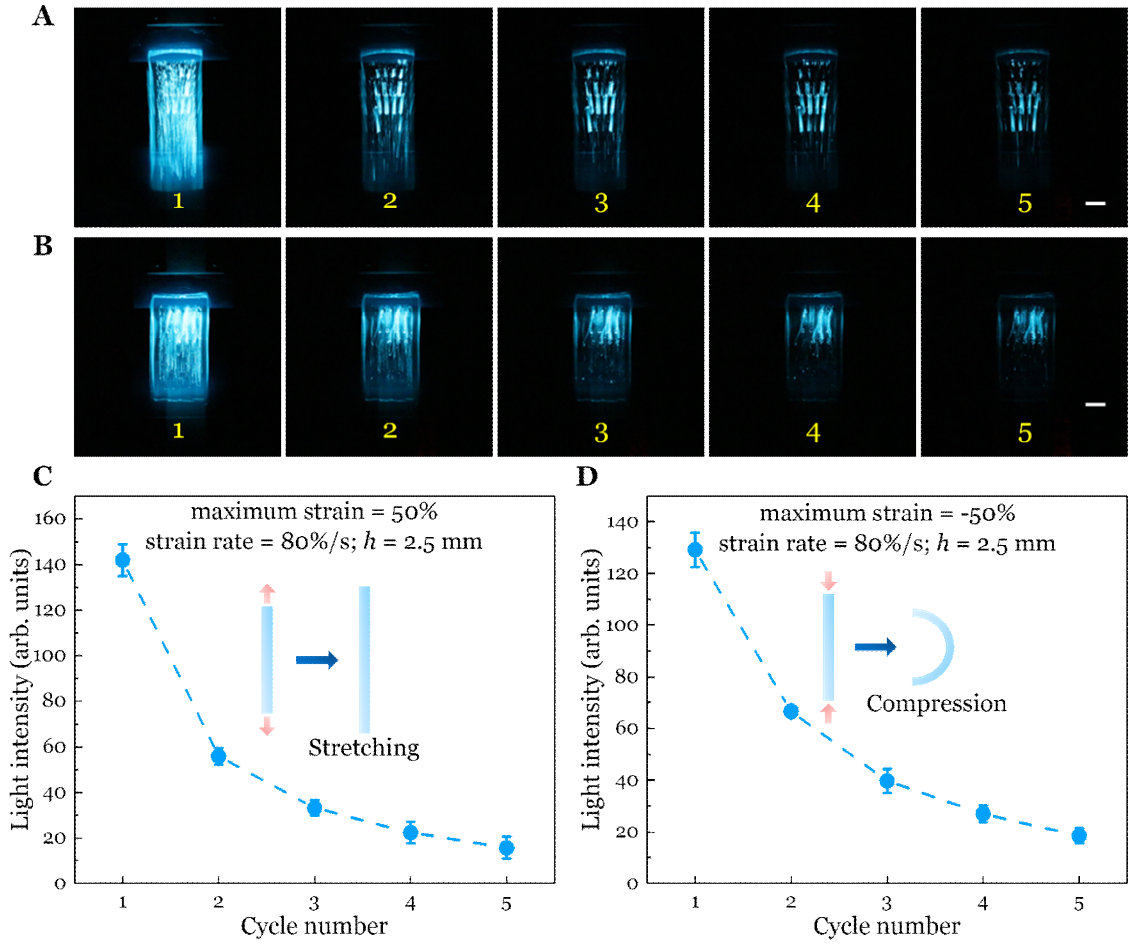

Supplementary Figure 12. Effect of cyclic loading-unloading stretching and compression on light intensity for the chamber with *h* = 2.5 mm pillars. (**A**) Bioluminescence within the biohybrid device stimulated by continuous stretching cycles. Numbers in each panel refer to cycle number, each 5 s in duration. Bioluminescence was brightest for the first stretching cycle, and then decreased for each subsequent cycle, as numbered. Scale bar, 1 cm (**B**) Bioluminescence was brightest for the first compression cycle, and then decreased for each subsequent cycle, as numbered. Scale bar, 1 cm. (**C**) Light intensity as a function of stretching cycle, for a maximum strain of 50% and strain rate of 80% s^{-1}, for chambers with h = 2.5 mm pillars. (**D**) Light intensity as a function of compression cycle, for a maximum strain of -50% and strain rate of 80% s^{-1} for chambers with h = 2.5 mm pillars. Values in (**C**) and (**D**) represent averages with standard deviations for N = 3 replicates.

Supplementary Note 2

Effect of Cyclic Loading-Unloading Stretching and Compression on Light Intensity

We explored the effect of cyclic loading-unloading stretching and compression on light intensity using the chamber with h = 2.5 mm pillars (Supplementary Fig. 12). The maximum strain and strain rate were set as 50% (or -50%) and 80% s^{-1}, respectively. Light intensity decreased exponentially with cycle number for both stretching (Supplementary Fig. 12A, C) and compression (Supplementary Fig. 12B, D). This result was consistent with the first flash of *Pyrocystis spp*. being the brightest, with each successive flash decreasing in intensity[1, 2].

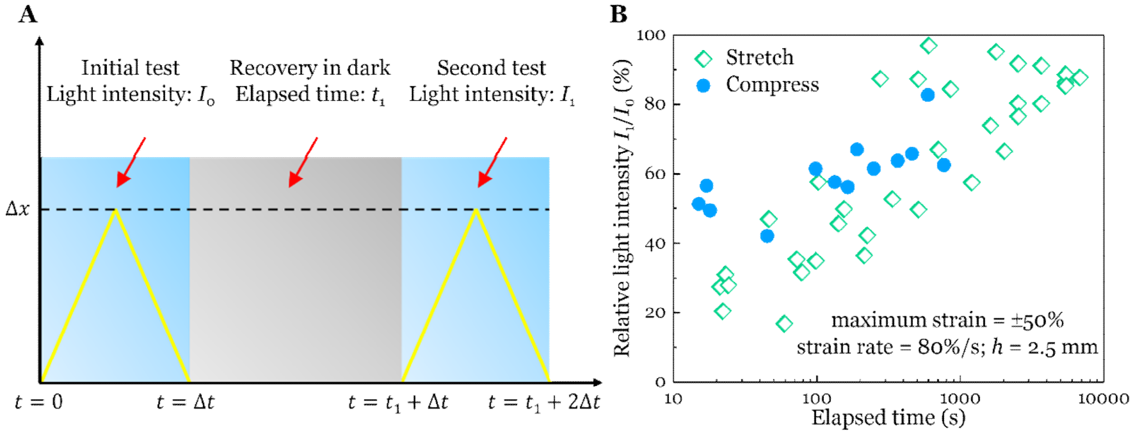

Supplementary Figure 13. Test protocol and results for bioluminescence recovery within the biohybrid device. (**A**) Initially, we applied one cycle of the triangle loading-unloading to the biohybrid device and recorded the light intensity as I_0. Afterwards, the device remained in darkness for different times t_1 to allow recovery of bioluminescence. Then, we applied the same test to the device and recorded the light intensity as I_1. The recovery ability is defined as the relative light intensity I_1/I_0. (**B**) Relative light intensity as a function of elapsed time after initial stretching or compression, for a maximum strain of 50% (or -50%) and strain rate of 80% s^{-1} for chambers with 2.5 mm high pillars.

Supplementary Note 3

Recovery of Bioluminescence of the Biohybrid Devices

To further study the recovery ability of bioluminescence of the biohybrid devices, we applied one cycle of the triangle loading-unloading with maximum strain as 50% (or -50%) and strain rate as 80% s^{-1} to the device and recorded the light intensity as I_0. Then, the device was placed in darkness to allow the bioluminescence system to recover. After different time intervals, we then applied a second cycle of stretching or compression and measured the light intensity as I_1, with the relative light intensity defined as I_1/I_0 (Supplementary Fig. 13A). Relative light intensity increased with the elapsed time for both stretching and compression (Supplementary Fig. 13B), consistent with the recovery of bioluminescence measured for *P. fusiformis*, which takes 30 min for full recovery[2].

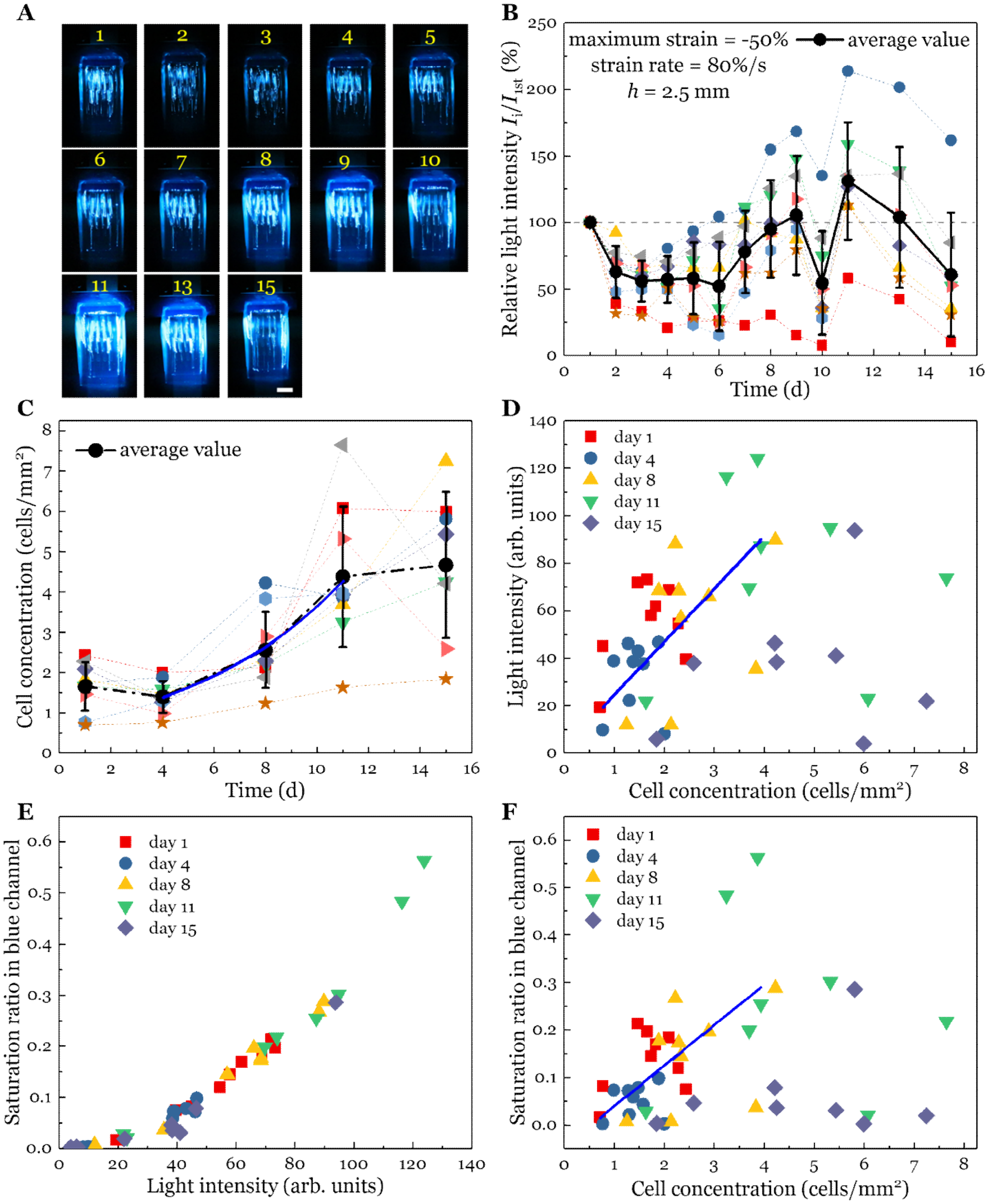

Supplementary Figure 14. Change of bioluminescence and cell concentration within the biohybrid devices under the same compression test for 15 continuous days. (**A**) Representative change of bioluminescence of a biohybrid device for 15 continuous days. Numbers in each photo refer to time (day). Scale bar, 1 cm. (**B**) Light intensity induced by compression as a function of time after initial testing. The biohybrid devices were bathed in seawater and maintained on a light:dark cycle in between tests. Each device is represented by a different symbol, with the average represented in the black circle. (**C**) The change of cell concentration during the experiment. Each color/symbol represents the results for each of the 9 devices. The black symbol and dash dot line

represents the average cell concentration of all devices. The solid blue curve represents the exponential regression for days 4, 8 and 11 only (R^2 = 0.99). The slope of the regression is 0.16 day^{-1}, which represents the population growth rate. (**D**) Light intensity increased with cell concentration up to 3.9 cells mm^{-2}, with the blue line representing the linear regression for that range (R^2 = 0.45). (**E**) The saturation ratio in the blue channel of the RGB image increased with light intensity based on grayscale analysis. (**F**) The saturation ratio in the blue channel of the RGB image increased with cell concentration up to 3.9 cells mm^{-2}, with the blue line representing the linear regression for that range (R^2 = 0.35). Values in (**B**) and (**C**) represent averages with standard deviations for N = 9 replicates.

Supplementary Note 4
Viability of the Biohybrid Devices

We characterized the viability of the biohybrid devices by measuring the light intensity under the same loading and also measuring the concentration of cells inside the devices for 15 continuous days (Supplementary Fig. 14). Sufficiently bright bioluminescence of a biohybrid device was maintained throughout this period (Supplementary Fig. 14A). Relative light intensity was defined as I_i/I_{1st}, where I_{1st} and I_i are the light intensity of the first day and subsequent day i, respectively. The average relative light intensity for 9 devices initially decreased to ~56% by day 3, where it was maintained through day 6, after which it increased to ~131% by day 11, and finally decreased to ~61% by day 15 (Supplementary Fig. 14B). The decrease of light intensity on day 10 by all devices was attributed to inadvertent pre-stimulation of the device during the experimental handling. Thus, our biohybrid devices were able to maintain a high relative light intensity (>55% of initial values) for at least 15 days, indicating high viability.

The pattern of bioluminescence was due to changes in average cell concentration (Supplementary Fig. 14C), which slightly decreased from 1.7 cells mm^{-2} on day 1 to 1.4 cells mm^{-2} on day 4, indicating cell mortality as confirmed by the presence of dead cells. From day 4 through day 11, the average cell concentration increased exponentially to 4.4 cells mm^{-2}, with a growth rate of 0.16 day^{-1}, corresponding to a doubling time ln(2)/0.16 of 4.3 days, which is consistent with previous studies of dinoflagellate growth[3, 4]. This pattern was generally consistent with the change of relative light intensity (Supplementary Fig. 14B). The average cell concentration increased slightly by day 15, although the growth rate was no longer exponential. In contrast, during this period there was a decrease in relative light intensity, suggesting a change in cell physiological state. Light intensity increased as a function of cell concentration for the day 4-11 period, but only for concentrations up to 3.9 cells mm^{-2} (Supplementary Fig. 14D), showing a similar pattern to the results in Supplementary Fig. 8D. The saturation ratio in the blue channel increased with light intensity (Supplementary Fig. 14E), but with higher values than that in Supplementary Fig. 8E. Meanwhile, the saturation ratio in the blue channel increased with cell concentration up to 3.9 cells mm^{-2} (Supplementary Fig. 14F), showing a pattern similar to the results in Supplementary Fig. 8F. The results of this experiment suggest that days 4-11 are optimal for cell growth and light emission measurements.

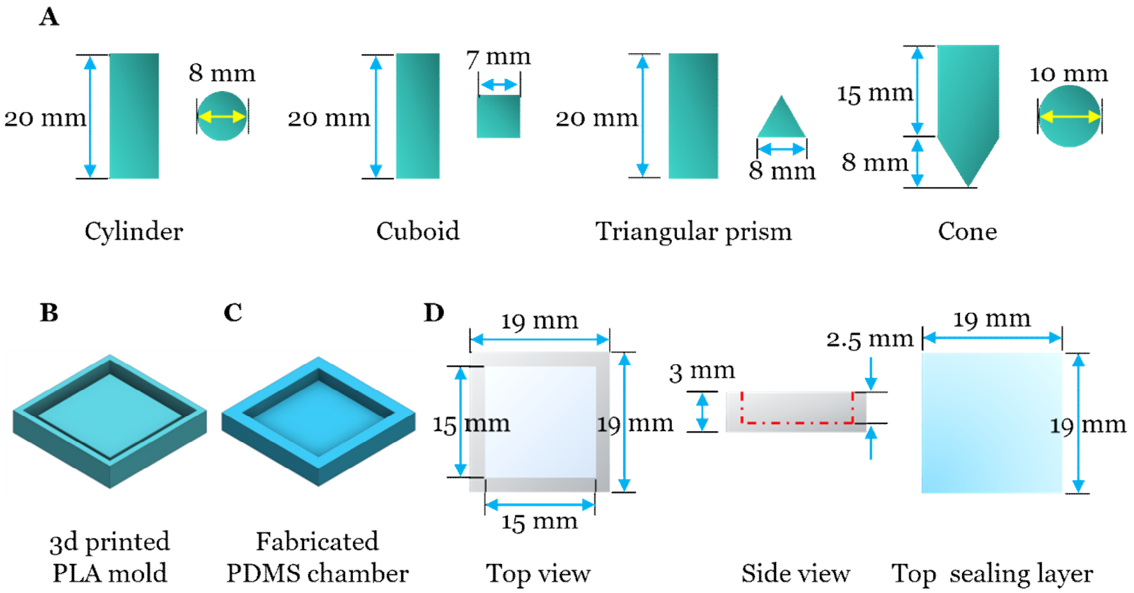

Supplementary Figure 15. Description of the objects with various geometries and the single chamber biohybrid device. (**A**) Dimensions of the cylinder, rectangular block, triangle prism, and cone. (**B**) Schematic of the 3D printed PLA mold. (**C**) Schematic of the molded PDMS chamber. (**D**) Dimensions of the molded PDMS chamber in (C), for top and side views, and dimensions of the top sealing layer.

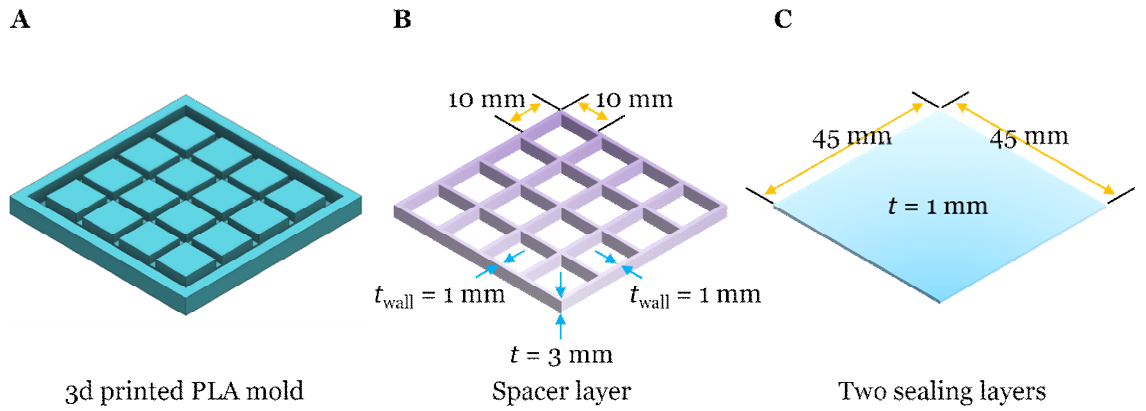

Supplementary Figure 16. Description of the 4×4 multichambered biohybrid device. (**A**) Schematic of the 3D printed PLA mold for making the spacer layer. (**B**) Dimensions of the molded spacer layer. (**C**) Dimensions of the two transparent sealing layers.

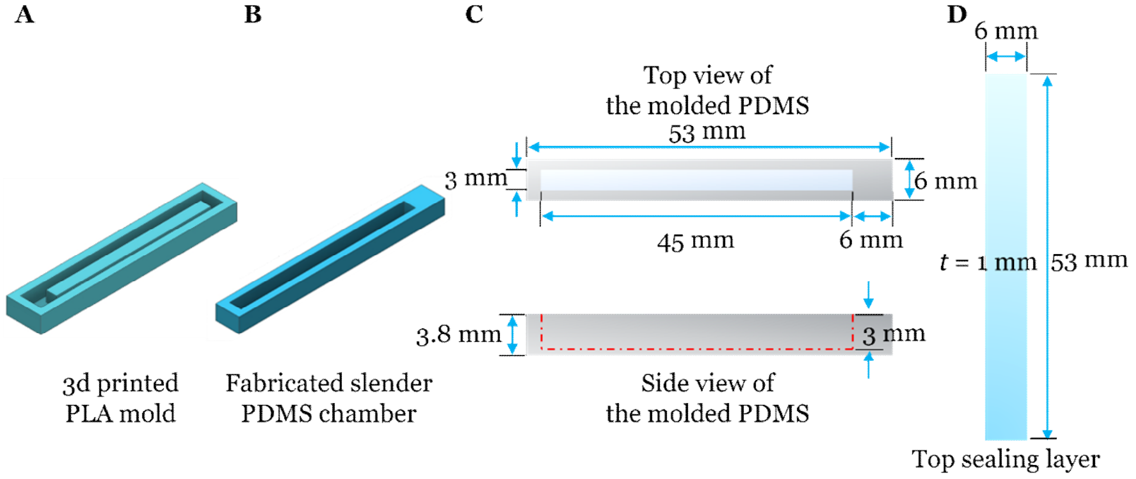

Supplementary Figure 17. Description of the slender biohybrid device for detecting airflow. (**A**) Schematic of the 3D printed PLA mold. (**B**) Schematic of the molded slender PDMS chamber. (**C**) Schematics of top and side views of the device showing the dimensions of the molded PDMS chamber in (B). (**D**) Dimensions of the top sealing layer.

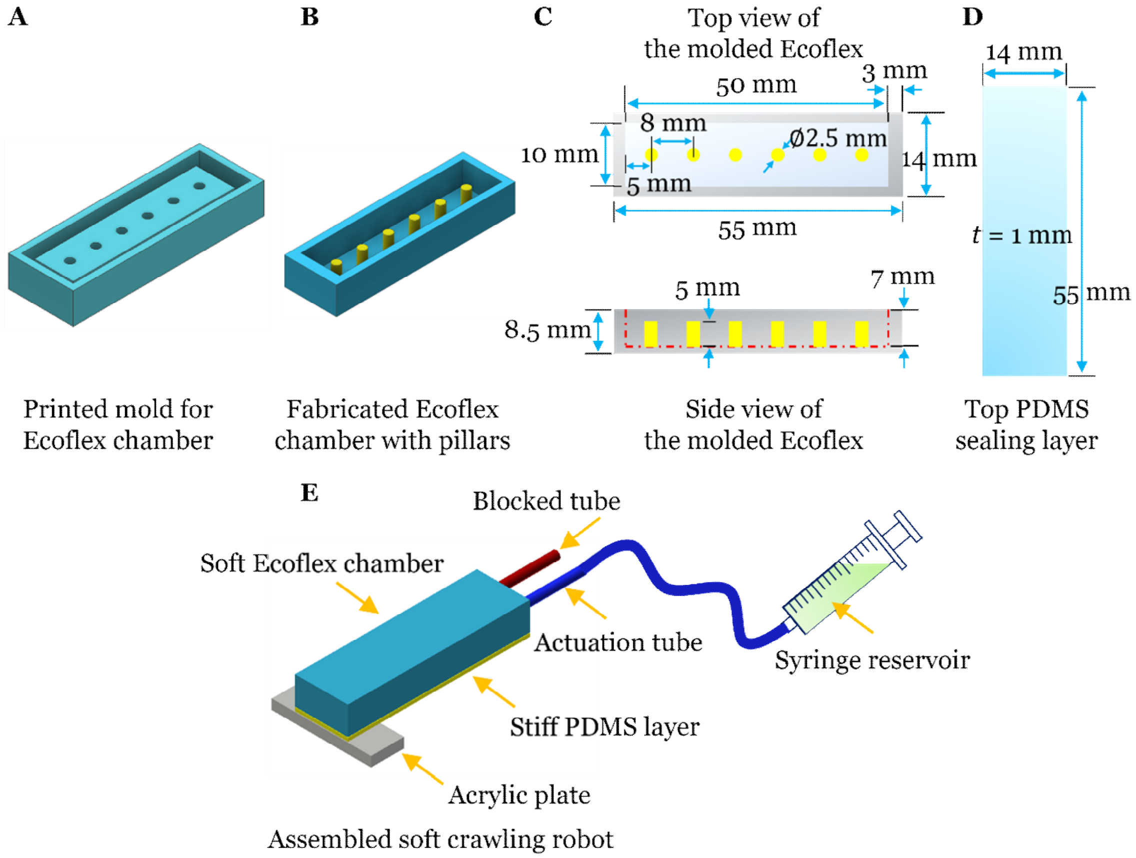

Supplementary Figure 18. Description of the hydraulic actuated soft crawling robot. (**A**) Schematic of the 3D printed PLA mold. (**B**) Schematic of the molded Ecoflex chamber. (**C**) Schematics of top and side views showing the dimensions of the molded Ecoflex chamber in (B). (**D**) Dimensions of the top sealing layer. (**E**) The soft robot was built by assembling the Ecoflex chamber and the stiff PDMS layer. A piece of acrylic plate was attached to the front of the robot to induce friction difference on the substrate. Two tubes were connected to the robot for hydraulic actuation. The robot was first filled with dinoflagellate culture solution and then one tube was blocked. The other actuation tube was connected to a syringe reservoir of dinoflagellate culture solution.

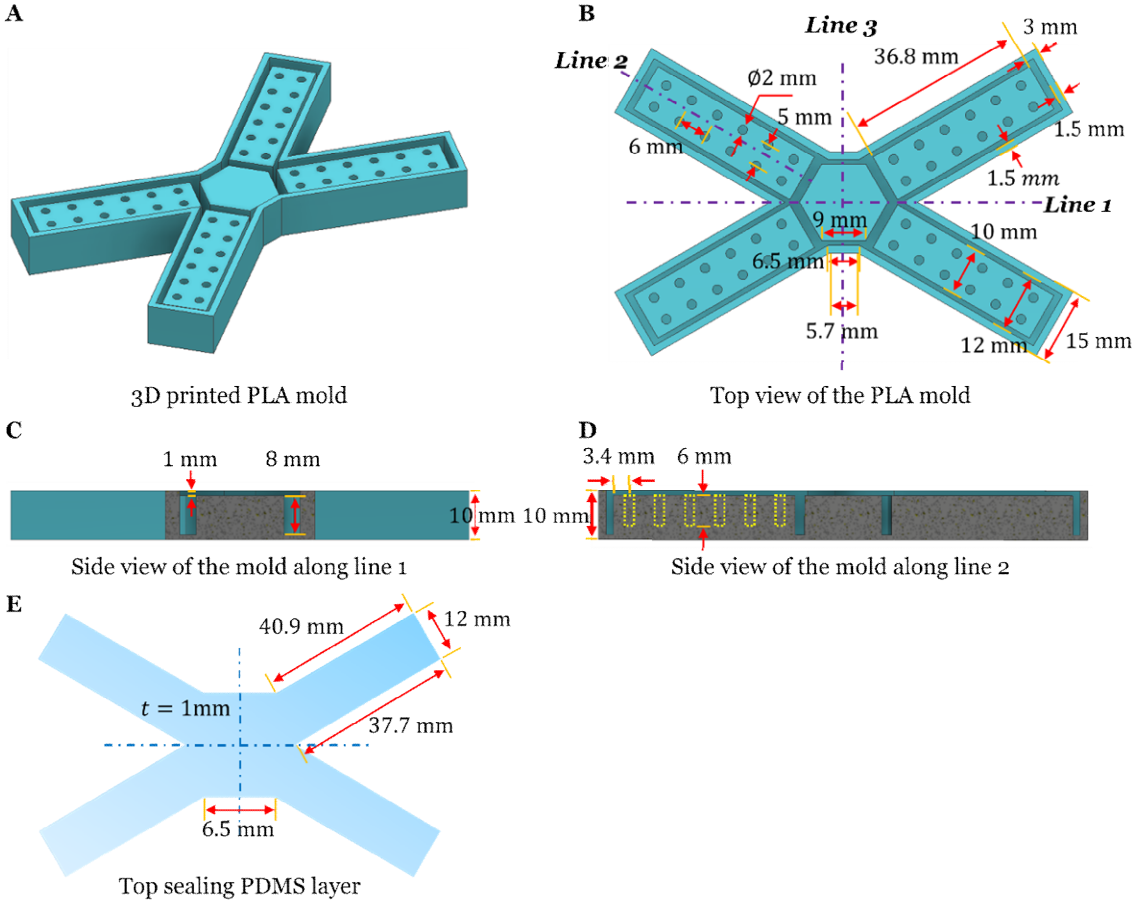

Supplementary Figure 19. Description of the untethered tetrapod-like soft robot with four legs. (**A**) Schematic of the 3D printed PLA mold for fabricating the Ecoflex chamber with pillars. (**B**) Top view schematic showing dimensions of the mold in (A). (**C**) Side view schematic and dimensions of the mold in (A) along line 1. (**D**) Side view schematic and dimensions of the mold in (A) along line 2. (**E**) Dimensions of the top sealing PDMS layer.

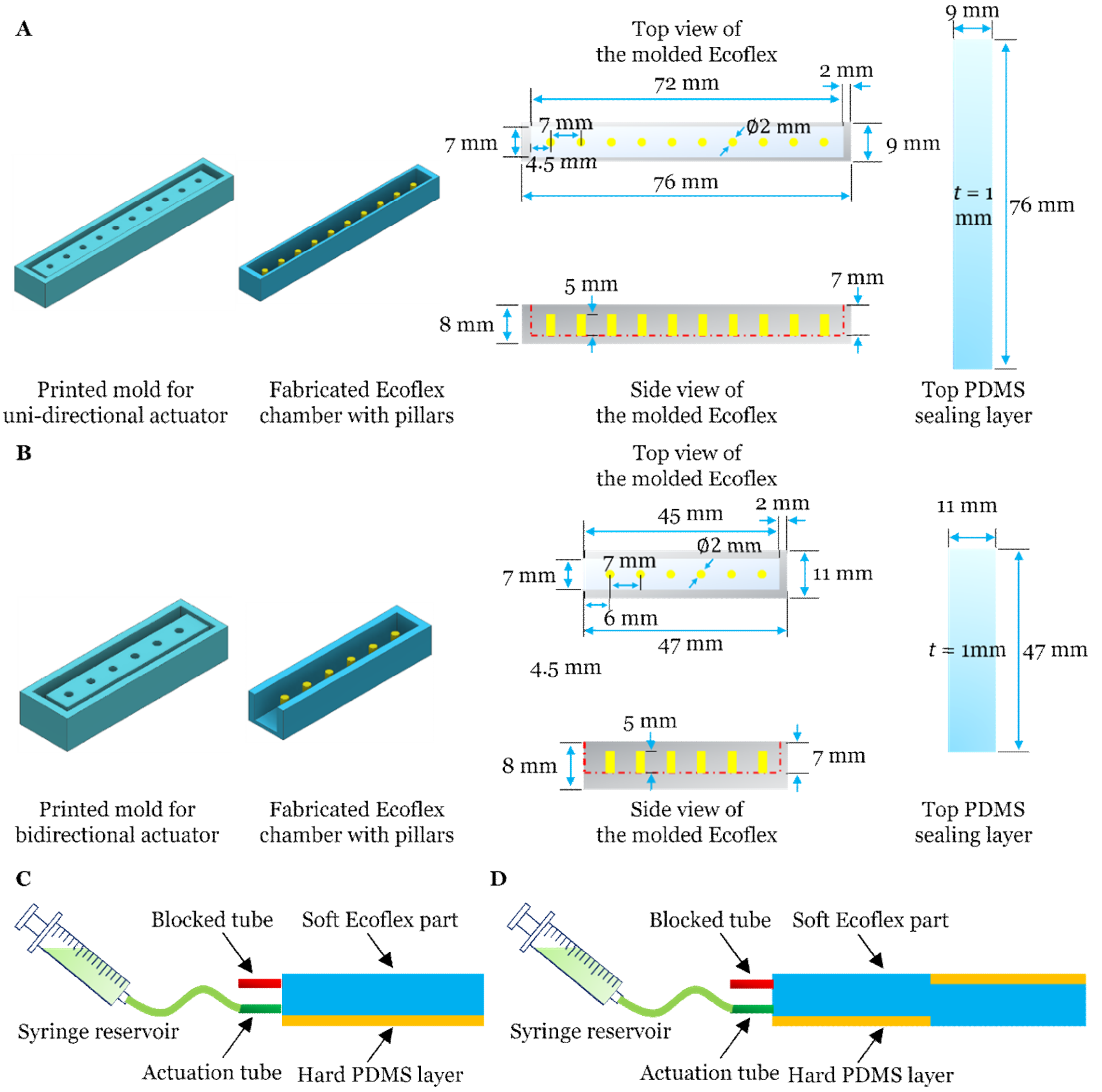

Supplementary Figure 20. Description of the hydraulic actuated soft uni-directional and bidirectional bending actuators. (**A**) Schematic of the 3D printed PLA mold, the molded Ecoflex chamber, and the dimensions of the molded Ecoflex chamber and the top sealing layer for the uni-directional bending actuator. (**B**) Schematic of the 3D printed PLA mold, the molded Ecoflex chamber, and the dimensions of the Ecoflex chamber and the top sealing layer for the bidirectional bending actuator. (**C**) The assembled uni-directional bending actuator. (**D**) The assembled bidirectional bending actuator.

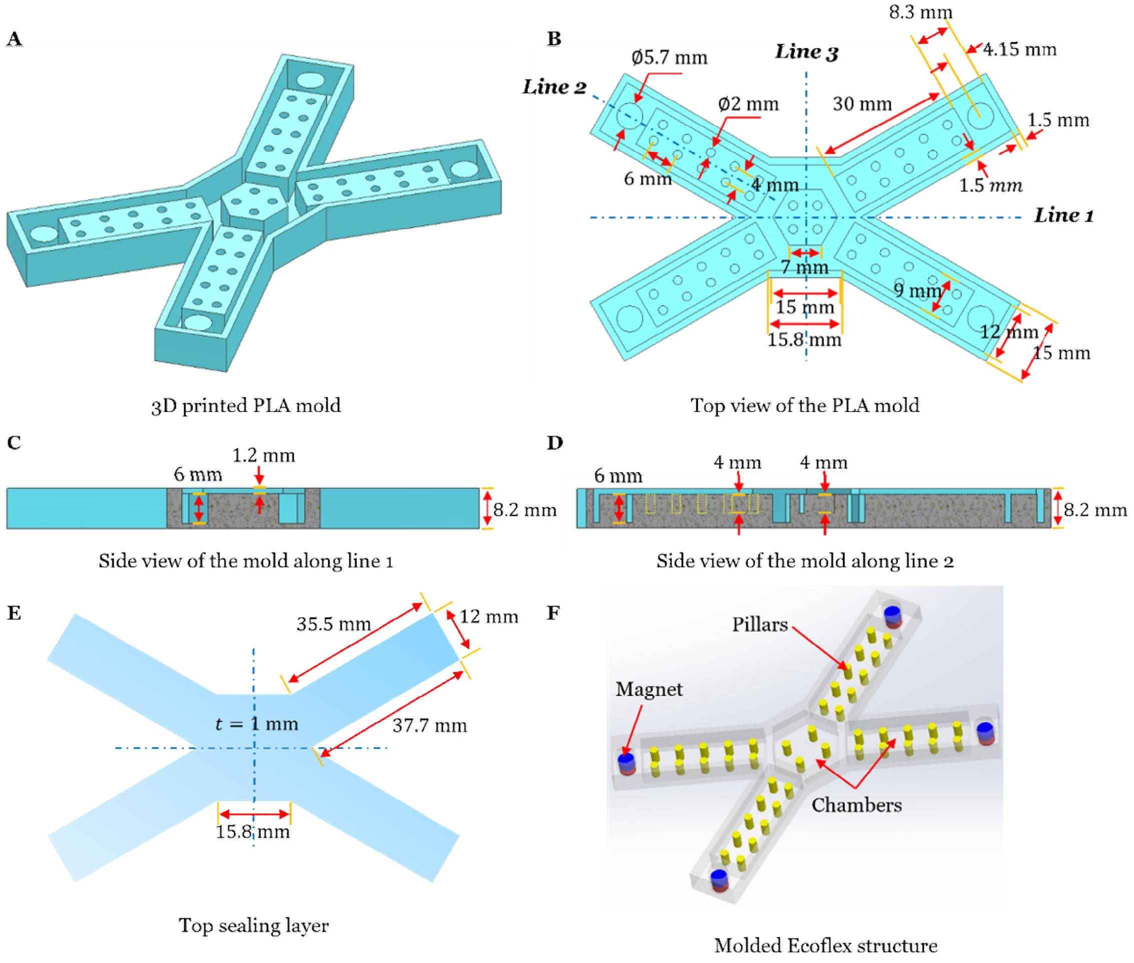

Supplementary Figure 21. Description of the untethered magnetically controlled soft robot. (**A**) Schematic of the 3D printed PLA mold for fabricating the Ecoflex chamber. (**B**) Top view schematic showing dimensions of the mold in (A). (**C**) Side view schematic and dimensions of the mold in (A) along line 1. (**D**) Side view schematic and dimensions of the mold in (A) along line 2. (**E**) Dimensions of the top sealing layer. (**F**) Schematic of the molded Ecoflex structure, consisting of five chambers in the center and four legs. Cylindrical pillars were introduced into each chamber to enhance fluid motion under magnetic actuation. Then a permanent magnet was embedded into the tip of each leg. Finally, the molded Ecoflex chamber and the sealing layer were glued together and then infused with culture solution to form the biohybrid soft robot.

Supplementary References

1. Tesson, B. & Latz, M. I. Mechanosensitivity of a rapid bioluminescence reporter system assessed by atomic force microscopy. *Biophys. J.* **108**, 1341-1351 (2015).
2. Widder, E. A. & Case, J. F. Two flash forms in the bioluminescent dinoflagellate, *Pyrocystis fusiformis*. *J. Comp. Physiol.* **143**, 43-52 (1981).
3. Sullivan, J. M. & Swift, E. Effects of small-scale turbulence on net growth rate and size of ten species of marine dinoflagellates. *J. Phycol.* **39**, 83-94 (2003).
4. Latz, M. I., Allen, J., Sarkar, S. & Rohra, J. Effect of fully characterized unsteady flow on population growth of the dinoflagellate *Lingulodinium polyedrum*. *Limnol. Oceanogr.* **54**, 1243-1256 (2009).
