# Supplementary material for: Highly robust and soft biohybrid mechanoluminescence for optical signaling and illumination
Source: Nat Commun. 2022 Jul 7;13:3914. doi: 10.1038/s41467-022-31705-6 (PMC9263131; doi:10.1038/s41467-022-31705-6)
Supplement: Supplementary file 3 — Description of Additional Supplementary Files [file 41467_2022_31705_MOESM3_ESM.pdf]

**Description of Additional Supplementary Files**

File Name: Supplementary Movie 1
Description: Bioluminescence of the dinoflagellate *Pyrocystis lunula* stimulated by shaking

File Name: Supplementary Movie 2
Description: Side view of the bioluminescence of the dinoflagellate *Pyrocystis lunula* stimulated by the vibration of a cantilever.

File Name: Supplementary Movie 3
Description: Top view of the bioluminescence of the dinoflagellate *Pyrocystis lunula* stimulated by the vibration of colored cantilevers.

File Name: Supplementary Movie 4
Description: Bioluminescence of the dinoflagellate *Pyrocystis lunula* within a device stimulated by contact by objects of different geometries.

File Name: Supplementary Movie 5
Description: Active actuation-induced and passive disturbance-induced bioluminescence of the soft actuator/robot for illumination.

File Name: Supplementary Movie 6
Description: Actuation-induced bioluminescence of the uni-directional and bidirectional soft bending actuators for optical signaling.

File Name: Supplementary Movie 7
Description: Actuations of the four-legged soft robot in different sequences to display programmable patterns for optical signaling.

File Name: Supplementary Movie 8
Description: Bioluminescence of the dinoflagellate *Pyrocystis lunula* due to magnetic actuation of an untethered biohybrid soft robot.

File Name: Supplementary Movie 9
Description: Maintenance of bioluminescence of the magnetically controlled biohybrid soft robot.
